# Social cognition in autism: Face tuning

**DOI:** 10.1038/s41598-017-02790-1

**Published:** 2017-05-26

**Authors:** Marina A. Pavlova, Michele Guerreschi, Lucia Tagliavento, Filippo Gitti, Alexander N. Sokolov, Andreas J. Fallgatter, Elisa Fazzi

**Affiliations:** 1 Department of Biomedical Magnetic Resonance, Medical School, Eberhard Karls University of Tübingen, Tübingen, Germany; 2 Department of Clinical and Experimental Sciences, University of Brescia, Brescia, Italy; 3 Unit of Child and Adolescent Neurology and Psychiatry, Asst Spedali Civili di Brescia, Brescia, Italy; 4 Women’s Health Research Institute, Department of Women’s Health, Medical School and University Hospital, Eberhard Karls University of Tübingen, Tübingen, Germany; 5 Department of Psychiatry and Psychotherapy, Medical School and University Hospital, Eberhard Karls University of Tübingen, Tübingen, Germany

## Abstract

Faces convey valuable information for social cognition, effective interpersonal interaction, and non-verbal communication. Face perception is believed to be atypical in autism, but the origin of this deficit is controversial. Dominant featural face encoding is suggested to be responsible for face tuning scarcity. Here we used a recently developed Face-n-Food paradigm for studying face tuning in individuals with autistic spectrum disorders (ASD). The key benefit of these images is that single components do not explicitly trigger face processing. In a spontaneous recognition task, adolescents with autism and typically developing matched controls were presented with a set of Face-n-Food images in different degree resembling a face (slightly bordering on the Giuseppe Arcimboldo style). The set of images was shown in a predetermined order from the least to most resembling a face. Thresholds for recognition of the Face-n-Food images as a face in ASD individuals were substantially higher than in typically developing controls: they did not report seeing a face on the images, which controls easily recognized as a face, and gave overall fewer face responses. This outcome not only lends support to atypical face tuning, but provides novel insights into the origin of face encoding deficits in autism.

## Introduction

Faces possess a special status across different domains of cognitive functioning due to their social relevance: they convey valuable information for effective interpersonal interaction and non-verbal communication. Many neuropsychiatric, neurodevelopmental, and psychosomatic disorders are characterized by impairments in visual social cognition, body language reading, and facial assessment of a social counterpart^1–5^ that may lead to interpersonal awkwardness. Face processing is widely believed to be atypical in autism^6–12^.

Autism spectrum disorders (ASD) represent a range of neurodevelopmental conditions characterized by impairment in social interaction and communication, co-occurring with restricted interests and repetitive behaviors, such as persistent fixations on parts of objects^13^. The disorder has a wide spectrum in severity of symptoms, intelligence quotient (IQ) level, performance on cognitive tasks, and brain neuroanatomy. ASD individuals display diminished orientation towards faces or face disinclination, impaired eye contact, and other deficits in face processing and recognition. Yet the origin of these impairments is still poorly understood, and the experimental evidence is controversial^8, 14^. It is postulated that ASD individuals preferentially exhibit featural face encoding. They are biased toward detailed facial information over the global form processing, better recognizing isolated facial cues and inverted faces than typically developing (TD) peers^12, 15, 16^. Autistic individuals prefer single face elements, in particular, located in the lower part of the face such as mouth^15, 17–19^, whereas for TD individuals, eyes play an important role in face decoding and recognition. Individuals with autism fail to engage in the emotionally or socially relevant content of social scenes by devoting substantially more time to the area of mouth than to eyes^20^. Face identity discrimination in autism is more difficult when access to local cues is minimized, and when dependence on integrative analysis is increased^21^. Other studies demonstrate, however, deficits of ASD individuals in configural face processing (for review, see ref. 8): they point to the substantial face inversion effect in ASD along with intact sensitivity to the Thatcher illusion^22, 23^ (a perceptual phenomenon indicating that in typical development, display inversion severely weakens configural face processing. This illusion is named after the late former British Prime Minister Margaret Thatcher, on whose photograph the inversion effect was first demonstrated by Peter Thompson in 1980^24^).

Face tuning is an automatic, rapid and primarily subconscious process, constituting one of the core components of the social perception^25^. Faces can be easily seen in non-face images such as grilled toasts, clouds or landscapes^26^. This phenomenon reflects high tuning to faces termed *face pareidolia*. TD infants are reported to be well tuned to pareidolic faces, i.e., protofaces or schematic faces^27^. Studies with face-like non-face objects indicate that TD children aged 24–60 months are more likely to direct their first fixation towards upright face-like objects than ASD individuals that points to poor face orientation and tuning in autism^28^. Yet high functioning adolescents with ASD are as sensitive to faces as TD peers^29^. Most recently, however, it had been shown that ASD children aged 8–18 years identify substantially fewer depictions of face-like objects as faces in a sequence of ambiguous stimuli^30^. Both adolescents and adults with ASD showed preferential detection of upright protofaces (schematic faces) under continuous flash suppression stimuli^31, 32^ (CFS, a technique with a target presented to one eye rendered invisible by high-contrast masks flashed into the other eye; ref. 33). Under this condition, visual stimuli are suppressed from awareness, and cortical face processing is strongly reduced, whereas subcortical brain areas continue to respond to invisible stimuli.

**Figure 1:**
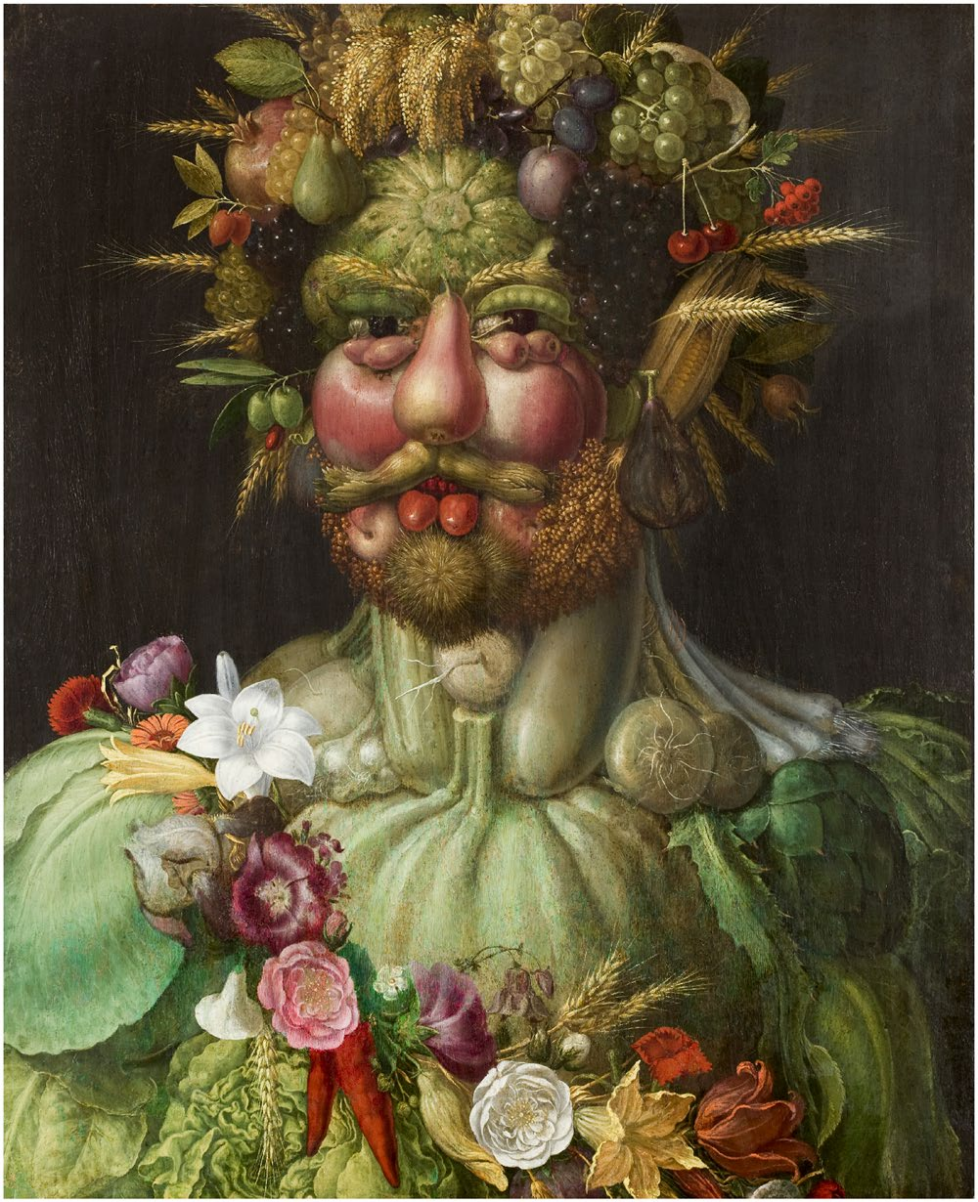
Example of the Giuseppe Arcimboldo style. The painting ‘Vertumnus’ by Guiseppe Arcimboldo (1526–1593), an Italian painter best known for creating fascinating imaginative portraits composed entirely of fruits, vegetables, plants, and flowers, depicts the Holy Roman emperor Rudolf II as Vertumnus, the Roman God of metamorphoses (http://vangoyourself.com/paintings/vertumnus; *public domain*).

**Figure 2:**
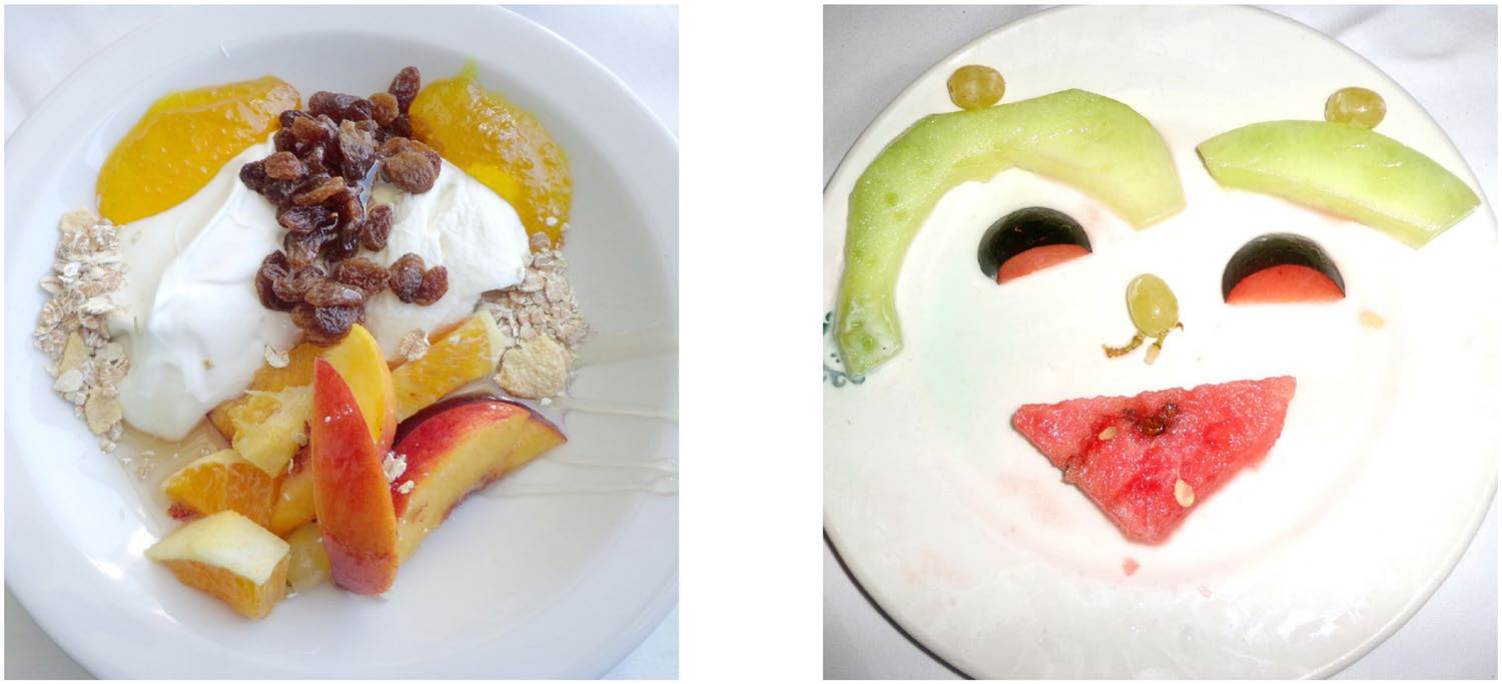
Examples of images from the Face-n-Food task. The least resembling face (left panel) and most resembling face (right panel) images from the Face-n-Food task (from Pavlova MA, Scheffler K, Sokolov AN. 2015. Face-n-Food: Gender Differences in Tuning to Faces. PLoS ONE 10(7): e0130363. doi:10.1371/journal.pone.0130363; the Creative Commons Attribution (CC BY) license).

The present work was aimed at investigation of face tuning in individuals with ASD in a recently created Face-n-Food task^34^. This task consists of a set of food-plate images composed of food ingredients (fruits, vegetables, sausages, etc.) in a manner slightly bordering on the style of Giuseppe Arcimboldo (1526–1593), an Italian painter best known for creating imaginative portraits composed entirely of fruits, vegetables, plants, flowers, books, and even body parts^35, 36^ (Figs 1 and 2). Obviously, one can perceive a Face-n-Food image either as a composition of elements (fruits, vegetables, etc.) or as a Gestalt (a face). As mentioned earlier^34^, the primary advantage of these images is that single components do not explicitly trigger face-specific processing, whereas in face images commonly used for investigating face perception (such as photographs or depictions), the mere occurrence of typical features or cues (such as a nose or mouth) already implicates face presence. For individuals with ASD, the use of such images provides an additional benefit of not being confounded by social features present in real faces, notably the eyes: the eye region of the face is perceived by ASD individuals as socially threatening, and elicits an increased physiological response as indicated by heightened skin conductance and amygdala activity^14^. In addition, in the Face-n-Food task, face tuning occurs spontaneously without being explicitly cued. We assume, therefore, that if in autistic individuals face tuning is deficient because of weakened configural face processing, they would experience more difficulties on the Face-n-Food task than TD controls. This task also benefits from using unfamiliar ‘face’ images that is of importance in clinical settings^37^. Tuning to faces in the Arcimboldo paintings emerges early in perceptual development: already infants aged 7–8 months prefer the Arcimboldo portraits over the same images presented ‘wrong way up’^38^. On overall, TD adults and children possess an entire bias for seeing faces in Arcimboldo-like images.

## Results

**Figure 3:**
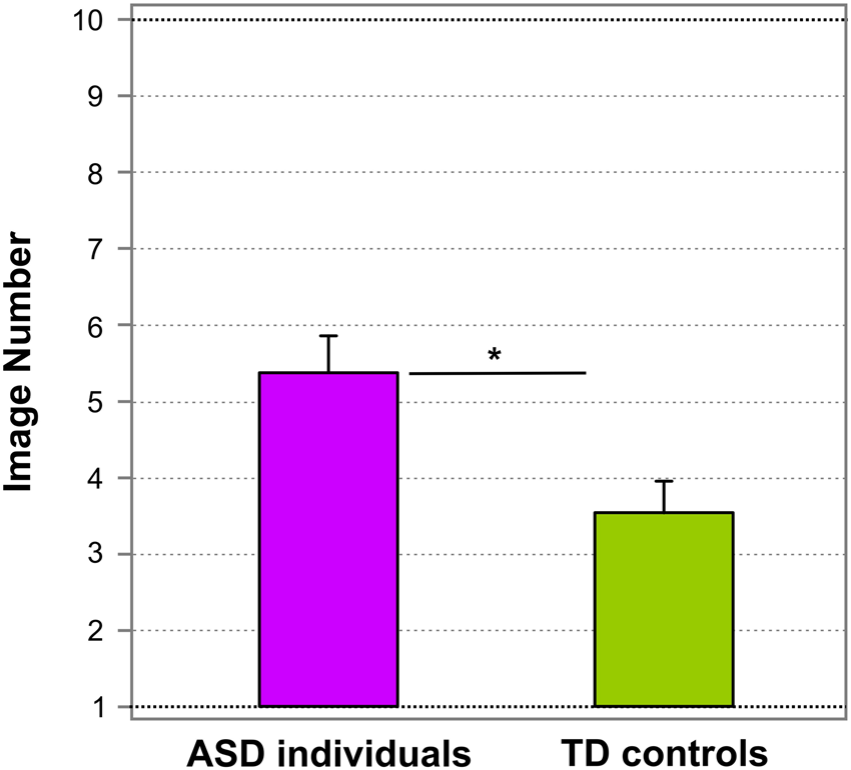
Tuning to faces. The average image number, on which resembling a face on the Face-n-Food task (face response) was initially reported, separately for ASD individuals and TD controls. Vertical bars represent SEM. Significant difference in thresholds for face tuning between ASD individuals and TD controls is indicated by asterisk.

Participants were presented with the set of Face-n-Food images, one by one, in the predetermined order from the least to most resembling a face (images 1 to 10). Both TD and ASD individuals either described a food-plate image in terms of food composition (non-face response, 0) or as a face (face response, 1). Figure 3 shows the thresholds for face tuning (i.e., average image number, on which face response was initially reported on the Face-n-Food task) separately for ASD individuals and TD controls. ASD individuals experienced more troubles in spontaneous recognition of the images as a face: TD controls reported seeing a face on average on 3.56 ± 1.59 (mean ± SD) image, whereas ASD individuals (14 out of 16) gave the first face response on average on 5.36 ± 1.91 image. Two out of 16 ASD individuals completely failed on the Face-n-Food task: they did not spontaneously recognize even the most recognizable image number 10 as a face. ASD individuals significantly differed from matched TD controls on the face recognition thresholds (t(28) = 2.25, p < 0.016, two-tailed, with an effect size Cohen’s d = 1.02).

Once seen as a face, Arcimboldo paintings are often processed with a strong face-dominating bias. However, both ASD and TD individuals delivered non-face responses on some subsequent Face-n-Food images. As some participants did not report seeing a face on all subsequent images after an initial face report, an additional analysis was performed on the total number of face responses. The difference in the overall percentage of face responses between TD (70.63 ± 16.52; mean ± SD) and ASD individuals (48.13 ± 25.09) was significant (t(30) = 2.74, p < 0.005, two-tailed, with an effect size Cohen’s d = 1.06).

**Figure 4:**
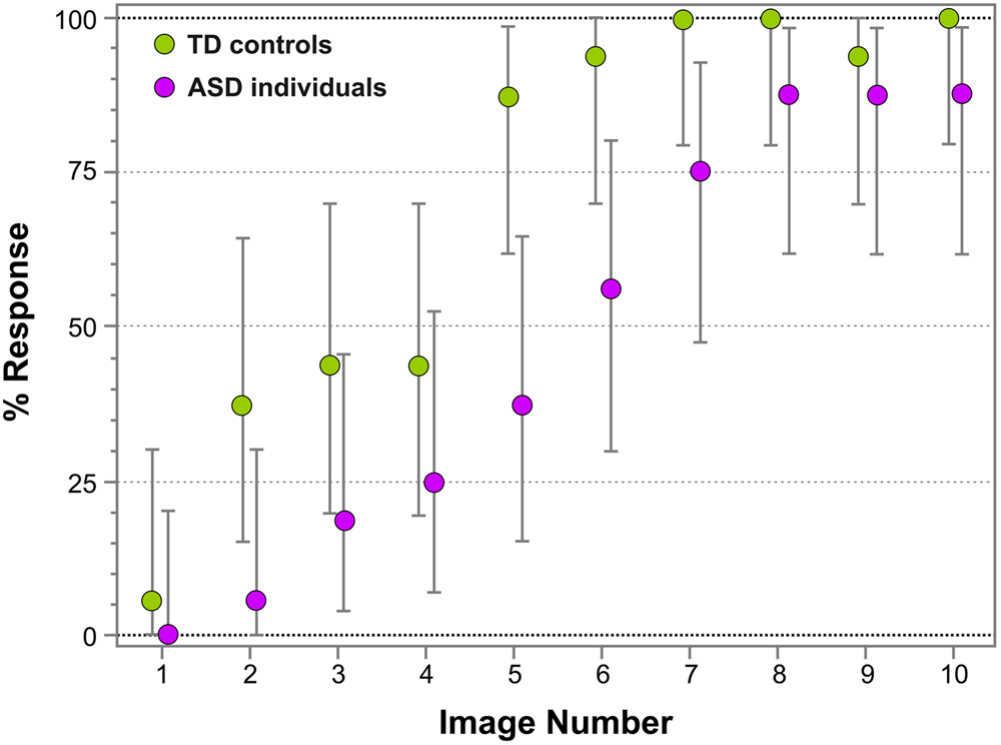
Percentage of face responses for ASD individuals and TD controls. The image number reflects its face resemblance (1 – the least recognizable, 10 – the most recognizable as a face). Vertical bars represent 95% CI.

Figure 4 represents the percentage of face responses for each Face-n-Food image for ASD individuals and TD controls. As seen from this figure, individuals with ASD much later reported seeing a face and gave on overall much fewer face responses. As indicated by multiple stepwise nominal logistic regression analysis, the effect of group (TD vs. ASD) is significant (χ^2^(1) = 24.9, p < 0.0001). For the first five images, ASD individuals provided far less than 50% of face responses, whereas TD group gave almost 50% face responses already from the third image. Starting from the images 5–6, controls very fast reached the ceiling level of performance. By contrast, ASD individuals much later than controls attained the maximal number of face responses for their group, and still gave only 87.7% face responses even with the most resembling face image number 10. Although face recognition level of ASD individuals is much lower, there is no significant interaction between group and image number (χ^2^(9) = 6.77, p = 0.661, n.s.): the spontaneous face recognition is uniformly shifted down in ASD individuals as compared to TD controls.

**Figure 5:**
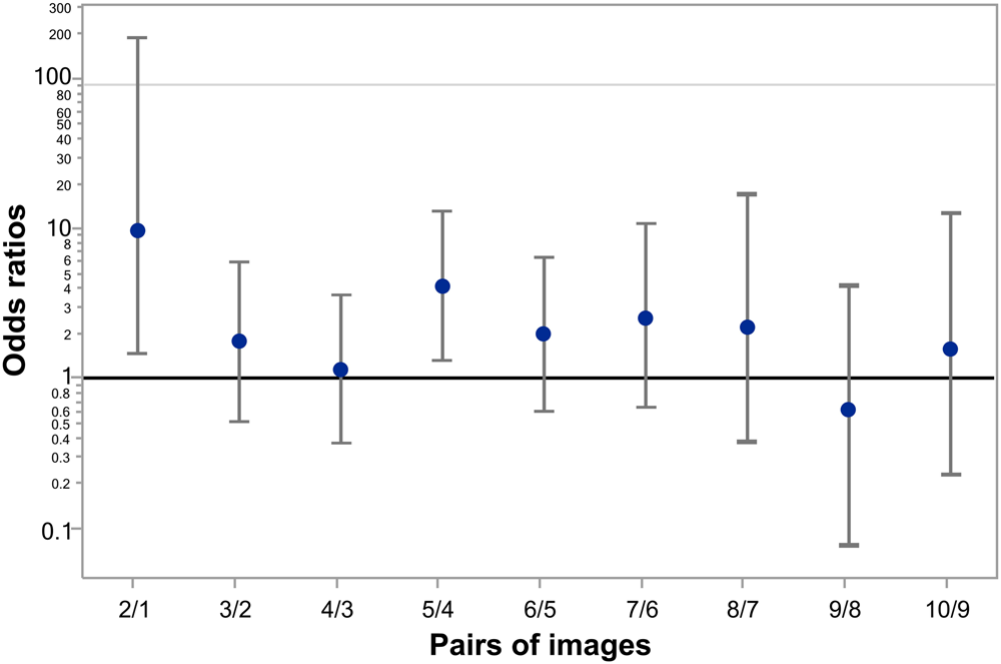
Odds ratios of face recognition between pairs of Face-n-Food images. Vertical bars represent 95% CI. The significant leaps in face recognition occur from the image 1 to 2 and image 4 to 5. The odds ratios for all other pairs do not differ from 1, indicating the lack of increase in face recognition.

Figure 5 represents odds ratios for all consecutive pairs of Face-n-Food images independent of group. The leaps in face recognition occurred from the image 1 to 2 with an odds ratio of 6.07 (95% CI, confidence interval, 0.005 to 0.658; p < 0.01) and from the image 4 to image 5 with an odds ratio 6.24 (95% CI, 0.075 to 0.742; p < 0.01). The odds ratios for all other pairs (3/2, 4/3, 6/5, 7/6, 8/7, 9/8, and 10/9) are not significantly greater than 1 that indicates the lack of substantial increase in face recognition. As shown by the likelihood ratio analysis, in TD controls as compared to ASD individuals, the odds ratio to give face response to each Face-n-Food image in the set is 6.38 (95% CI, 3.21 to 13.6; p < 0.0001).

**Figure 6:**
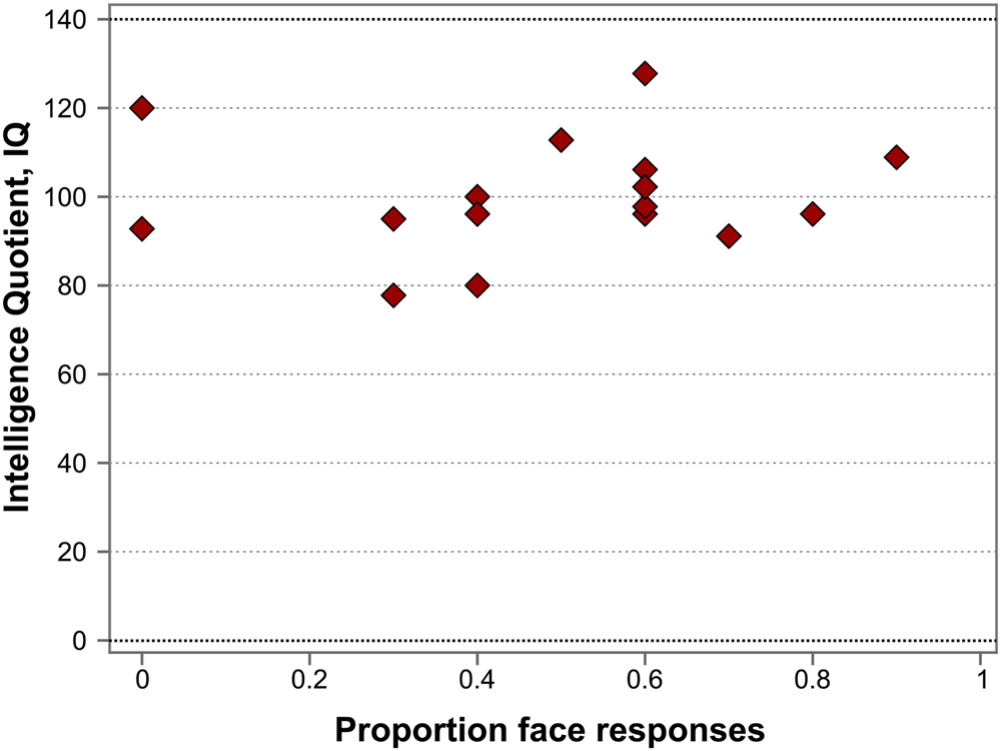
Face resemblance and the general IQ scores in ASD individuals. No substantial link occurs between face resemblance (as proportion of face responses) and the IQ scores.

In ASD individuals, no correlation was found between their performance on the Face-n-Food task (face response rate) and the general IQ scores (Spearman’s rho = 0.239, p = 0.37, n.s., two-tailed; Fig. 6). This indicates that the impaired performance on the Face-n-Food task in ASD individuals stems from the face tuning deficits rather than from general cognitive disabilities that may affect task performance.

## Discussion

By using the recently created Face-n-Food task consisting of a set of food-plate images that comprised food ingredients such as fruits, vegetables, and sausages^34–36^, we investigated face tuning in autism. The key benefit of these images is that their single components do not explicitly trigger face processing. The findings indicate that ASD individuals exhibit poor face tuning on the Face-n-Food task. Thresholds for recognition of the Face-n-Food images as a face in ASD individuals were substantially higher than in TD matched controls: they did not report seeing a face on the images, which TD matched controls easily recognized as a face, and gave on overall fewer face responses.

The most plausible explanation for this outcome is that eficits on the Face-n-Food task are triggered by difficulties of ASD individuals in visual feature integration: one can perceive a Face-n-Food image either as a composition of elements (fruits, vegetables, etc.) or as a whole or Gestalt (a face). Once seen as a face, the Face-n-Food images are processed with a strong face-dominating bias and, therefore, top-down influences may substantially affect bottom-up visual processing of these images. In line with this, recent findings in TD adults indicate that original Arcimboldo hidden-face portraits are judged as being more ambiguous by perceivers with local as compared with global perceptual style^39, 40^.

Recently, the Face-n-Food paradigm was used for face tuning examination in the other neurodevelopmental disorder, namely, in individuals with Williams-Beuren syndrome (WS)^36^. Strikingly, although WS individuals possess a hypersocial personality profile that is manifested as a drive for social interaction and particular face fascination, their tuning to faces is extremely poor. As compared to individuals with WS, the sample of ASD individuals appears to be less impaired on the Face-n-Food task. Although both groups differ in respect to gender, cultural background, and age of participants (WS individuals in Pavlova *et al*.^36^ were aged 23.3 ± 10.6 years with age range 8 to 44 years, whereas ASD individuals in the present study were aged 14.13 ± 1.86 years with age range 11 to 17; chronological age can be considered as one of predictors of featural face decoding in autistic individuals, but less in WS individuals^41^), it appears that WS individuals experience more troubles in spontaneous recognition of the images as a face. The threshold of face tuning on the Face-n-Food task is much higher in WS as compared to ASD individuals (8.18 ± 1.47 vs 5.36 ± 1.91 image, respectively). Most interesting, as indicated by the form and slopes of the fitted face recognition curves (Fig. 4 of this paper and Fig. 4 of Pavlova *et al*.^36^), the recognition dynamics is remarkably different. Although both groups gave about 85% face responses even on the image 10 most resembling a face, WS individuals did not recognize the first five images as a face at all, and were close to 50% face recognition even on image 9, whereas in ASD individuals, face recognition continuously elevated resulting in face recognition above 50% already on the images 6–7, and reached the ceiling level for their group on images 8–10. This means that face tuning scarcity in ASD and WS individuals may be of diverse origin. Clarification of the precise nature of this deficit is of immense value. Our assumption about the diverse origin in face processing impairment dovetails well with the electroencephalographic (EEG) findings. Differences in EEG gamma band oscillatory brain activity (that is thought to underlie visual binding of elements) suggest that, although both ASD and WS individuals tend to rely more on featural processing in face recognition, the precise machinery of featural processing differs between the two disorders: In autism, apparently normal bursts of gamma activity occur, but they are rather similar for upright and inverted faces, whereas in WS individuals, no clear gamma peaks are observed for both upright and inverted faces^42^.

To date, there is no consensus on whether face encoding abilities are preserved in autism. The evidence on the origin of face encoding deficits in ASD remains controversial, with some arguments for typical holistic processing and other arguments for atypical development with a preference for featural encoding^8, 41^. The fusiform face area (FFA), a region known to be heavily involved in typical face processing, is less or differently activated in autism^43–46^. Moreover, lower functional connectivity of the FFA with the frontal cerebral cortex had been reported^46^. Other indispensable parts constituting the social brain such as the superior temporal sulcus (STS) and amygdala, which is heavily involved in affective processing with and without awareness^47^, also exhibit atypical activation in autism^48–51^. In ASD, EEG shows atypical face-specific N170 component of the event-related potentials (ERPs) with bilateral, as compared to typically right lateralized, voltage distribution^52^. Yet functional magnetic resonance imaging (fMRI) studies found normal FFA activation when gaze patterns and attention to faces, and notably to the eyes, were controlled^48, 53^. It is also assumed that aberrant face encoding in ASD primarily stems from an eye-avoidance strategy resulting from the eye region of the face being perceived as socially threatening^14^. In ASD individuals, positive correlation is found between amygdala activation and gaze time spent in the region of eyes^44^. The present work suggests a limited ability of ASD individuals for seeing faces in the Face-n-Food images, and may be considered as a further step towards putting the Face-n-Food task into clinical setting. One possible explanation for this outcome is that the Face-n-Food test may be more sensitive to preferences for the featural/local face encoding strategy.

Only a few brain imaging studies in TD individuals investigated brain response to Arcimboldo-like images, and the outcome of these studies appears controversial. In the occipito-temporal network underpinning face processing (including the FFA), bilateral superior and inferior parietal cortices, and the right inferior frontal gyrus, Arcimboldo portraits compared to Renaissance portraits and non-artistic face representations (photographs) elicit greater fMRI response^39^. When contrasting with the same upside-down paintings, Arcimboldo portraits activate the right FFA and posterior STS^54^, two essential parts of the social brain. In the right hemisphere, face-sensitive N170 component of the ERP is the same in response to Arcimboldo portraits and natural faces, whereas in the left hemisphere N170 amplitude is larger for natural faces^55^. In 7–8 month-olds infants, near-infrared spectroscopy indicates that the left temporal area is more responsive to the Arcimboldo portraits than to single elements (such as vegetables) constituting these images^38^.

As mentioned earlier^36^, important step in further elaborating the Face-n-Food task would be recording of the functional brain activity. Comparison of topographic patterns and temporal dynamics of the neural circuitry underpinning facial processing (with hubs in the FFA and posterior STS, which are considered pivots of the social brain) between individuals with diverse neurodevelopmental disorders such as ASD and WS individuals can add essential information on typical and atypical face processing.

In the light of profound non-face visual perceptual deficits in ASD^56^, it is essential to figure out whether face processing has a special status in ASD individuals. In the present study, face tuning on the Face-n-Food task does not relate to general cognitive abilities as measured by general IQ. This indicates that the poor performance on this task in ASD individuals does not relate to possible intellectual or cognitive disability. In other words, the impaired task performance in ASD most probably stems from face tuning as opposed to a number of other alternative explanations.

As the only one female participant with ASD had been enrolled in this work, one of the study’s limitations is that it left beyond investigation possible sex differences in autism. It is well-known that males have a higher risk for developing ASD than females, with a sex ratio of about 4:1^57^ or even higher. Yet females are affected much more severely, and therefore, in high-functioning autistic individuals, this ratio may be higher. Furthermore, females possess higher risk of under-identification of autism due to a possible ‘female camouflage effect’^58^. Taken together, this suggests that in clinical settings, access to female ASD individuals is more difficult. The lack of studies in females with autism calls for a thorough investigation of their neurobiological profile^2^. Neuroanatomy of autism differs between females and males^59^. Structural MRI reveals sex-specific morphology in young children (aged 2–7 years) with autism: in boys with ASD, two male-specific regions of increased gray matter volume (the left middle occipital gyrus, BA 19, and right superior temporal gyrus, BA 22) are reported, whereas in girls with ASD, increased grey matter volumes are found in the bilateral frontal regions, right anterior cingulate cortex (BA 32), and the right cerebellum^60^. Studies investigating gender/sex differences in face encoding in autism are extremely sparse. It is reported that face identity and face recognition are stronger impaired in autistic males^61, 62^. In autistic girls, but not boys, atypical face-sensitive N170 component of the ERP is associated with symptom severity^63^. In the light of profound gender effects on the Face-n-Food task in TD adults^34, 35^, it is worthwhile for future research to take a close look at potential gender impact on face tuning in ASD.

## Resume

The outcome of this work indicates that autistic individuals exhibit substantial deficits in seeing faces in the Face-n-Food images represented by a composition of food ingredients in a manner bordering on the Giuseppe Arcimboldo style. In autism, thresholds for recognition of the Face-n-Food images as a face are substantially higher: they did not report seeing a face on the images, which TD matched controls easily recognized as a face. This outcome not only lends support for atypical face tuning, but provides novel insights into the origin of face encoding deficits in autism. The precise nature of this aberration including the brain mechanisms underlying face encoding and gender impact on this deficit remains to be clarified. In addition, comparison of face tuning in ASD and individuals with Williams syndrome^36^ suggests that face tuning scarcity in ASD and WS individuals may be of diverse origin.

## Methods

### Participants

Sixteen individuals with ASD (1 female, 15 males) were enrolled in the study. They were recruited at the Unit of Child and Adolescent Neurology and Psychiatry of Asst Spedali Civili (Civil Hospital) of Brescia, Italy. In addition to clinical expertise, autism^13^ was diagnosed using the Autism Diagnostic Interview - Revised (ADI-R)^64^ and Autistic Diagnostic Observation Schedule - General (ADOS-G)^65^. Participants were aged 14.13 ± 1.86 years (mean ± SD; age range, 11 to 17 years). Their general IQ (GIQ, WISC) was on average 100.06 ± 13.08 (ranging from 78 to 128, 14 of them had general IQ higher than 90). Most of them (12 out of 16) had severity level 1 (DSM-5), three out of 16 had level 2, and the only female participant had 3 (her general IQ was in the normal range, 95). Sixteen TD controls pairwise matched with ASD individuals for gender and age had been recruited from the local community of Brescia, Italy. Participants were run individually. All of them had normal or corrected-to-normal vision. None had previous experience with such images and tasks. The study was conducted in line with the Declaration of Helsinki and was approved by the local Ethics Committee of Asst Spedali Civili (Civil Hospital) of Brescia, Italy. Informed written consent was obtained from all participants or their care providers. Participation was voluntary, and the data were processed anonymously.

### The Face-n-Food task

The Face-n-Food task was administered to participants. This task is described in detail elsewhere^34–36^. For this task, a set of ten images was created that were composed of food ingredients (fruits, vegetables, sausages, etc.), and to different degree resembled faces. The images slightly border on the Giuseppe Arcimboldo style (Figs 1 and 2). Participants were presented with the set of images, one by one, in the predetermined order from the least to most resembling a face (images 1 to 10). This order was determined in the previous study with TD volunteers^34^, and had been used since once seen as a face, Face-n-Food images are often processed with a strong face-dominating bias. On each trial, participants had to perform a spontaneous recognition task: they were asked to briefly describe what they saw. Their reports were recorded, and then analyzed by independent experts. For further data processing, the responses were coded as either non-face (0) or face (1) report. No immediate feedback was provided. To avoid time pressure that can potentially cause stress and negative emotional and physiological reactions blocking cognitive processes, there was no time limit on the task. With each participant, the testing procedure lasted no longer than 20–25 min.

## References

[1] Pavlova MA (2012). Biological motion processing as a hallmark of social cognition. Cereb. Cortex.

[2] Pavlova MA (2017). Sex and gender affect the social brain: beyond simplicity. J. Neurosci. Res..

[3] Lazar SM, Evans DW, Myers SM, Moreno-De Luca A, Moore GJ (2014). Social cognition and neural substrates of face perception: implications for neurodevelopmental and neuropsychiatric disorders. Behav. Brain Res..

[4] Pelphrey KA, Yang DY, McPartland JC (2014). Building a social neuroscience of autism spectrum disorder. Curr Top Behav Neurosci.

[5] Feuerriegel D, Churches O, Hofmann J, Keage HA (2015). The N170 and face perception in psychiatric and neurological disorders: a systematic review. Clin. Neurophysiol..

[6] Jemel B, Mottron L, Dawson M (2006). Impaired face processing in autism: fact or artifact?. J. Autism Dev. Disord..

[7] Wolf JM (2008). Specific impairment of face processing abilities in children with autism spectrum disorder using the Let’s Face It! skills battery. J. Autism Res.

[8] Weigelt S, Koldewyn K, Kanwisher N (2012). Face identity recognition in autism spectrum disorders: a review of behavioral studies. Neurosci. Biobehav. Rev..

[9] Weigelt S, Koldewyn K, Kanwisher N (2013). Face recognition deficits in autism spectrum disorders are both domain specific and process specific. PLoS One..

[10] Dimitriou D, Leonard HC, Karmiloff-Smith A, Johnson MH, Thomas MS (2014). Atypical development of configural face recognition in children with autism, Down syndrome and Williams syndrome. J. Intellect. Disabil. Res..

[11] Koldewyn K, Jiang YV, Weigelt S, Kanwisher N (2013). Global/local processing in autism: not a disability, but a disinclination. J. Autism Dev. Disord..

[12] Tang J (2015). Face recognition and visual search strategies in autism spectrum disorders: amending and extending a recent review by Weigelt *et al*. PLoS One..

[13] American Psychiatric Association. *Diagnostic and Statistical Manual of Mental Disorders* (5^th^ Edition). Washington, DC. American Psychiatric Association (2013).

[14] Tanaka J, Sung A (2016). The ‘Eye Avoidance’ Hypothesis of Autism Face Processing. J. Autism Dev. Disord..

[15] Hobson RP, Ouston J, Lee A (1988). What’s in a face? The case of autism. Br. J. Psychol.

[16] Lahaie A (2006). Face perception in high-functioning autistic adults: evidence for superior processing of face parts, not for a configural face-processing deficit. Neuropsychology.

[17] Langdell T (1978). Recognition of faces: an approach to the study of autism. J. Child Psychol. Psychiatry.

[18] Klin A, Jones W, Schultz R, Volkmar F, Cohen D (2002). Visual fixation patterns during viewing of naturalistic social situations as predictors of social competence in individuals with autism. Arch. Gen. Psychiatry..

[19] Joseph RM, Tanaka J (2003). Holistic and part-based face recognition in children with autism. J. Child Psychol. Psychiatry.

[20] Rice K, Moriuchi JM, Jones W, Klin A (2012). Parsing heterogeneity in autism spectrum disorders: visual scanning of dynamic social scenes in school-aged children. J. Am. Acad. Child Adolesc. Psychiatry.

[21] Morin K (2015). Atypical Face Perception in Autism: A Point of View?. Autism Res.

[22] Rouse H, Donnelly N, Hadwin J, Brown T (2004). Do children with autism perceive second-order relational features? The case of the Thatcher illusion. J. Child Psychol. Psychiatry.

[23] Cleary L, Brady N, Fitzgerald M, Gallagher L (2015). Holistic processing of faces as measured by the Thatcher illusion is intact in autism spectrum disorders. Autism.

[24] Thompson P (1980). Margaret Thatcher: a new illusion. Perception.

[25] Palermo R, Rhodes G (2007). Are you always on my mind? A review of how face perception and attention interact. Neuropsychologia.

[26] Evritt, L. Pareidolia: Why we see faces in hills, the Moon and toasties. BBC News Magazine. Available at: http://www.bbc.com/news/magazine-22686500 (2013).

[27] Kato M, Mugitani R (2015). Pareidolia in infants. PLoS One..

[28] Guillon Q (2016). Intact perception but abnormal orientation towards face-like objects in young children with ASD. Sci. Rep.

[29] Akechi H, Kikuchi Y, Tojo Y, Osanai H, Hasegawa T (2014). Neural and behavioural responses to face-likeness of objects in adolescents with autism spectrum disorder. Sci. Rep.

[30] Ryan C, Stafford M, King RJ (2016). Brief Report: Seeing the Man in the Moon: Do Children with Autism Perceive Pareidolic Faces? A Pilot Study. J. Autism Dev. Disord..

[31] Shah P, Happé F, Sowden S, Cook R, Bird G (2015). Orienting Toward Face-Like Stimuli in Early Childhood. Child Dev..

[32] Akechi H (2015). Preferential awareness of protofacial stimuli in autism. Cognition.

[33] Tsuchiya N, Koch C (2005). Continuous flash suppression reduces negative afterimages. Nat Neurosci.

[34] Pavlova MA, Scheffler K, Sokolov AN (2015). Face-n-Food: Gender differences in tuning to faces. PLoS ONE.

[35] Pavlova MA, Mayer A, Hösl F, Sokolov AN (2016). Faces on her and his mind: female and likable. PLoS ONE.

[36] Pavlova MA, Heiz J, Sokolov AN, Barisnikov K (2016). Social cognition in Williams Syndrome: face tuning. Front. Psychol.: Emotion Science.

[37] Koelkebeck K (2015). Benefits of using culturally unfamiliar stimuli in ambiguous emotion identification: A cross-cultural study. Psychiatry Res..

[38] Kobayashi M (2012). Do infants recognize the Arcimboldo images as faces? Behavioral and near-infrared spectroscopic study. J. Exp.Child Psychol..

[39] Boccia M (2014). Why do you like Arcimboldo’s portraits? Effect of perceptual style on aesthetic appreciation of ambiguous artworks. Atten. Percept. Psychophys..

[40] Boccia M (2015). Do you like Arcimboldo’s? Esthetic appreciation modulates brain activity in solving perceptual ambiguity. Behav. Brain Res..

[41] Annaz D, Karmiloff-Smith A, Johnson MH, Thomas MS (2009). A cross-syndrome study of the development of holistic face recognition in children with autism, Down syndrome, and Williams syndrome. J. Exp. Child Psychol..

[42] Grice SJ (2001). Disordered visual processing and oscillatory brain activity in autism and Williams syndrome. NeuroReport.

[43] Pierce K, Müller RA, Ambrose J, Allen G, Courchesne E (2001). Face processing occurs outside the fusiform ‘face area’ in autism: evidence from functional MRI. Brain.

[44] Dalton KM (2005). Gaze fixation and the neural circuitry of face processing in autism. Nat. Neurosci..

[45] Schultz RT (2005). Developmental deficits in social perception in autism: the role of the amygdala and fusiform face area. Int. J. Dev. Neurosci..

[46] Koshino H (2008). fMRI investigation of working memory for faces in autism: visual coding and underconnectivity with frontal areas. Cereb. Cortex.

[47] Diano M, Celeghin A, Bagnis A, Tamietto M (2017). Amygdala response to emotional stimuli without awareness: facts and interpretations. Front Psychol.

[48] Hadjikhani N (2004). Activation of the fusiform gyrus when individuals with autism spectrum disorder view faces. Neuroimage.

[49] Baron-Cohen S, Belmonte MK (2005). Autism: a window onto the development of the social and the analytic brain. Ann. Rev. Neurosci..

[50] Pelphrey KA, Morris JP, McCarthy G (2005). Neural basis of eye gaze processing deficits in autism. Brain.

[51] Hadjikhani N, Kveraga K, Naik P, Ahlfors SP (2009). Early (M170) activation of face-specific cortex by face-like objects. Neuroreport.

[52] McPartland J, Dawson G, Webb SJ, Panagiotides H, Carver LJ (2004). Event-related brain potentials reveal anomalies in temporal processing of faces in autism spectrum disorder. J. Child Psychol. Psychiatry.

[53] Perlman SB, Hudac CM, Pegors T, Minshew N, Pelphrey KA (2011). Experimental manipulation of face-evoked activity in the fusiform gyrus of individuals with autism. Soc. Neurosci..

[54] Rossion B, Dricot L, Goebel R, Busigny T (2011). Holistic face categorization in higher order visual areas of the normal and prosopagnosic brain: toward a non-hierarchical view of face perception. Front. Hum. Neurosci.

[55] Caharel S (2013). Early holistic face-like processing of Arcimboldo paintings in the right occipito-temporal cortex: evidence from the N170 ERP component. Int. J. Psychophysiol..

[56] Frith U (2004). Emanuel Miller lecture: Confusions and controversies about Asperger syndrome. J. Child Psychol. Psychiatry.

[57] Newschaffer CJ (2007). The epidemiology of autism spectrum disorders. Ann. Rev. Publ. Health.

[58] Rynkiewicz A (2016). An investigation of the ‘female camouflage effect’ in autism using a computerized ADOS-2 and a test of sex/gender differences. Mol. Autism.

[59] Lai MC (2013). Biological sex affects the neurobiology of autism. Brain.

[60] Retico A (2016). The effect of gender on the neuroanatomy of children with autism spectrum disorders: a support vector machine case-control study. Mol. Autism.

[61] Rhodes G, Jeffery L, Taylor L, Ewing L (2013). Autistic traits are linked to reduced adaptive coding of face identity and selectively poorer face recognition in men but not women. Neuropsychologia.

[62] Halliday DW, MacDonald SW, Sherf SK, Tanaka JW (2014). A reciprocal model of face recognition and autistic traits: evidence from an individual differences perspective. PLoS One.

[63] Coffman MC, Anderson LC, Naples AJ, McPartland JC (2015). Sex differences in social perception in children with ASD. J. Autism Dev. Disord..

[64] Lord C (2000). The Autism Diagnostic Observation Schedule, Generic: A standard measure of social and communication deficits associated with the spectrum of autism. J. Autism Dev. Dis..

[65] Lord C, Rutter M, Le Couteur A (1994). Autism Diagnostic Interview-Revised: a revised version of a diagnostic interview for caregivers of individuals with possible pervasive developmental disorders. J. Autism Dev. Dis..

